# Urban-Rural Mental Health Disparities in China: The Neighborhood Effects

**DOI:** 10.1093/geroni/igaa057.316

**Published:** 2020-12-16

**Authors:** Chengming Han

**Affiliations:** Case Western Reserve University, Cleveland, Ohio, United States

## Abstract

This paper intended to explore the neighborhood effects on mental health disparities of urban and rural residents in Mainland China. Data were drawn from the CHARLS baseline (2011). The sample included 450 neighborhoods, with 3907 urban residents and 13,391 rural residents older than 45 years old. Multilevel model was used to determine the neighborhoods’ effects and individual effects on depressive symptom scores (CES-D). Independent variables included social activities, health status, and demographic characteristics. The result reveals three context effects of urban-rural neighborhoods: first, people living in urban communities reported better physical health, higher educational levels, and lower depressive symptoms than their rural counterparts. Second, people living in urban communities are more engaged in social activities than people living in rural villages. Third, the urban neighborhoods present more variations in depressive symptoms and social activities than the rural neighborhoods.

